# GABRD Accelerates Tumour Progression via Regulating CCND1 Signalling Pathway in Gastric Cancer

**DOI:** 10.1111/jcmm.70485

**Published:** 2025-03-27

**Authors:** Weibing Leng, Jun Ye, Zhenpeng Wen, Han Wang, Zhenyu Zhu, Xilin Song, Kai Liu

**Affiliations:** ^1^ Colorectal Cancer Center Sichuan University West China Hospital Chengdu Sichuan China; ^2^ Department of Medical Oncology Sichuan University West China Hospital Chengdu Sichuan China; ^3^ Department of Proctology Traditional Chinese Medicine Hospital of Longquanyi Chengdu Sichuan China; ^4^ West China School of Medicine Sichuan University Chengdu Sichuan China; ^5^ Department of Gastrointestinal Surgery Shandong Cancer Hospital and Institute, Shandong First Medical University and Shandong Academy of Medical Sciences Jinan Shandong China

**Keywords:** cyclin D1, gamma‐aminobutyric acid type A receptor, gastric cancer, neurotransmitter receptors

## Abstract

Neurotransmitters and their receptors were reported to be involved in tumour initiation and progression. However, little is known about their roles in gastric cancer (GC). Here, we first identified gamma‐aminobutyric acid type A receptor subunit delta (GABRD) as a novel oncogene in GC. GABRD was preferentially upregulated in GC tissues compared with adjacent normal tissues. High GABRD expression was significantly associated with poor survival prognosis. Knockdown of GABRD could markedly induce cell apoptosis and cell cycle arrest while repressing proliferation and migration in vitro, and suppress tumour growth in vivo. The results of transcriptomic analysis and Ingenuity pathway analysis (IPA) highlighted that cyclin D1(CCND1) was a potential downstream target. Immunohistochemistry results also indicated that CCND1 expression was associated with GABRD in GC. Functional experiments also confirmed that the role of GABRD in regulating proliferation, migration, invasion, and apoptosis was dependent on CCND1. Mechanically, further research confirmed that GABRD knockdown could induce p53‐dependent apoptosis through CCND1, and GABRD upregulated CCDN1 through inhibiting its ubiquitin‐mediated degradation. Overall, these findings uncover a role for the neurotransmitter receptor GABRD in regulating the proliferation and apoptosis of gastric cancer cells. Our present study provides novel insights into the mechanism of tumourigenesis in gastric cancer.

**Abbreviations**
CCND1: Cyclin D1
Co‐IP: Co‐Immunoprecipitation
GABRD: Gamma‐aminobutyric acid type A receptor subunit delta
GC: gastric cancer
IHC: immunohistochemical
IPA: Ingenuity pathway analysis
TCGA: The Cancer Genome Atlas

## 1 Introduction

In recent years, emerging data suggest the neurotransmitter/receptor axis emerged as an essential microenvironmental component in influencing diverse malignant phenotypes of human cancers [1]. Neurotransmitter receptors have been detected in many tumour tissues and exert regulative effects in cancer cell proliferation and dissemination [2, 3, 4, 5, 6]. Therefore, deciphering the functional mechanisms of the neurotransmitter/receptor axis in tumorigenesis is expected to pave the way for novel anti‐cancer therapy [7].

Gamma‐aminobutyric acid (GABA), known as the inhibitory neurotransmitter in the central nervous system, is also prevalent in peripheral endocrine organs, such as the pituitary, adrenal medulla, and gastrointestinal tract [8]. Beyond its traditional neurotransmission, GABA receptors exert regulatory effects on tumour cell proliferation and migration in varies tumours [3, 4, 5, 9, 10, 11]. Specifically, Gamma‐aminobutyric acid type A receptor (GABAA receptor) subunit delta (GABRD) is found to be closely related to cancer. Pan‐cancer analysis of over 600 tumour and adjacent normal tissue pairs from The Cancer Genome Atlas (TCGA) revealed significant GABRD overexpression in 89% of subjects [12]. In another TCGA‐based bioinformatic study, GABRD expression was significantly increased in colorectal cancer compared with adjacent normal tissues and was associated with poor overall survival [13, 14, 15, 16]. However, GABRD expression was significantly decreased in IDH wild‐type diffuse low‐grade gliomas (LGG) compared with that in IDH mutant tumours and was independently associated with longer OS in IDH WT LGG [17]. These results indicated GABRD might play a role in the differentiation of tumour cells and the functional mechanism of GABRD in cancer needs to be studied in specific cancer types.

To date, the functional roles of GABRD in gastric cancer remain largely unexplored. In this study, we first identified GABRD as a novel oncogene in gastric cancer and investigated its molecular mechanism in gastric cancer. Our work expanded current understanding of neurotransmitter‐related signalling in cancer and provided novel insights for the realm of gastric cancer research.

## 2 Materials and Methods

### 2.1 TCGA Database Bioinformatics Analysis

The GDC download tool was utilised to obtain RNAseq count files in bulk, while the clinical information of The Cancer Genome Atlas Stomach Adenocarcinoma (TCGA‐STAD) was downloaded through the TCGAbiolinks package in R studio. For data standardisation, the method of estimating dispersion in DESeq2 was employed. A quality control assessment was conducted via principal component analysis (PCA).

### 2.2 Tissue Microarray and Immunohistochemical Analysis

Tissue microarrays containing 94 paraffin‐embedded primary gastric adenocarcinoma specimens and 86 adjacent normal specimens were obtained from Shanghai Outdo Biotech Co. Ltd. (cat. no. HStmA180Su19). All human subjects provided informed consent, and West China Hospital Sichuan University and Shanghai Outdo Biotech Co. Ltd. Institutional Review Board approval was acquired for this study. All patients with gastric cancer underwent surgery between June 2010 and November 2010, and the last follow‐up was completed in July 2016. The follow‐up information included age, gender, tumour grade, number of lymph node metastases, and time to recurrence and death. The microarrays were incubated with the rabbit polyclonal anti‐human GABRD (Table S1). All core biopsies were independently reviewed by two pathologists, and expression levels were defined by the sum of the grades for the percentage of positive staining and intensity.

### 2.3 Cell Culture, Gene Knockout, or Overexpression Cell Models

Gastric cancer cell lines (AGS, MKN‐45, MGC‐803) and gastric mucosal cell line (GES1) were purchased from BeNa Culture Collection. GES1 cells were cultured in DMEM high‐sugar medium with 10% FBS; AGS cells in F12K medium with 10% FBS; MKN‐45 and MGC‐803 cells in RPMI‐1640 medium with 10% FBS. Three shRNA sequences were designed based on the GABRD or CCND1 gene sequences, respectively, and were cloned into the BR‐V108 lentivirus vector. The target sequences were provided in Table S2.

### 2.4 Real‐Time qPCR (RT‐qPCR) and Western Blotting

The primers used in RT‐qPCR are shown in Table S3.

Information on the relevant antibodies used in the Western blotting assay is provided in Table S1.

### 2.5 Colony Formation Assay

In the colony formation assay, cells were inoculated into six‐well plates at a density of 2 mL/well, with three wells per experimental group. The cells were cultured for 14 days. Cell colonies were soaked with 1 mL paraformaldehyde per well for 30 min and then stained with Giemsa staining (500 μL/well; Shanghai Dingguo, Shanghai, China) for 20 min. Finally, the colonies were photographed and counted with a fluorescence microscope (Olympus, Japan). Each experiment was performed in triplicate.

### 2.6 Co‐Immunoprecipitation (Co‐IP)

AGS cells were lysed to extract protein, and the protein concentration was determined by the BCA method. The 1.0 mg of protein was reacted with the reference antibody overnight at 4°C. The protein‐antibody complex was incubated with 20 μL beads at 4°C for 2 h. The protein‐antibody‐beads complex was cleaned three times with IP lysate, and then 20 μg was taken for western blotting.

### 2.7 Nude Mouse Tumourigenic Model and In Vivo Imaging

All mouse procedures were approved by and performed in accordance with the Animal Ethical and Welfare Committee of Sichuan University and followed ARRIVE guidelines. 4‐week‐old female BALB/c nude mice were purchased from Jiangsu Jixuan Yaokang Biotechnology Co. All nude mice were divided into 2 groups of 10 mice each. 1 × 10^7^ lentivirus‐infected AGS cells were injected subcutaneously into nude mice. Tumour volume was measured every 3 days. Tumour volume was calculated as follows: Tumour volume = π/6 × L × W × W, where L represents the long diameter and W represents the short diameter. Nude mice were euthanised after 19 days. Tumours were removed from the nude mice and weighed. For in vivo imaging, mice were injected intraperitoneally with 200 μL of 15 mg/mL fluorescein 7–8 min before being anaesthetised with isoflurane. Imaging was performed using the IVIS 200 imaging system and Living Image software version 3.0.4 (Xenogen, Hopkinton, MA, USA). After 5 s of exposure, the total flux to each animal's region of interest (ROI) was recorded as photons/s.

### 2.8 Statistical Analysis

All the data in the study were expressed as mean ± standard deviation (SD). The data were analysed with R software. A *t*‐test was used to analyse the statistical differences between the two groups, and a one‐way ANOVA was used to evaluate the statistical differences between the three groups and above.

## 3 Results

### 3.1 GABRD Was Highly Expressed in Gastric Cancer and Correlated With Tumour Progression and Poor Survival Prognosis

**FIGURE 1:**
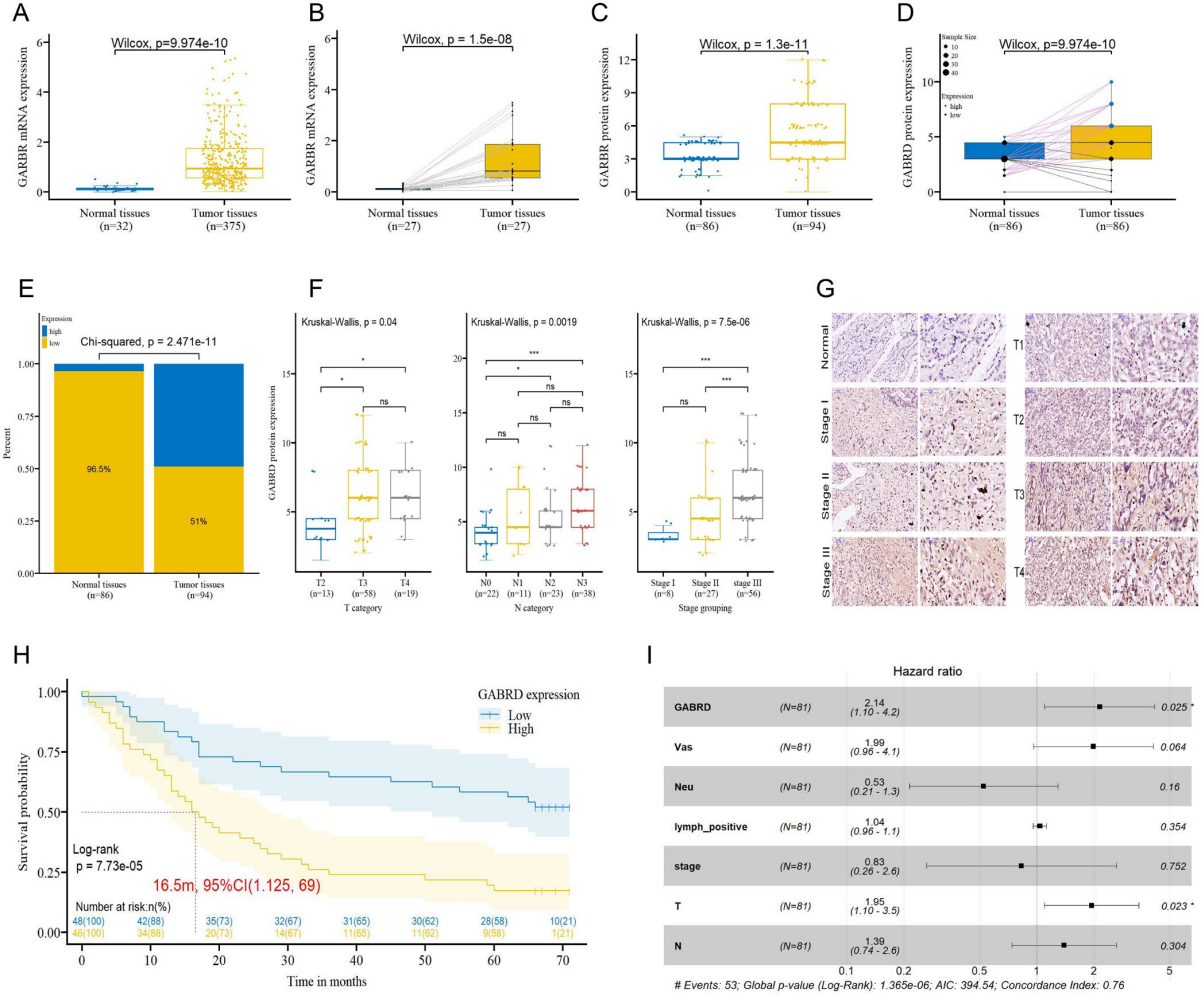
GABRD expression analysis in gastric cancer tissues and its prognostic implications. (A) GABRD mRNA expression levels in stomach adenocarcinoma tissues versus normal tissues from TCGA database. (B) Comparison of GABRD mRNA expression in 27 pairs of gastric cancer tissues and adjacent normal tissues. (C) Immunohistochemical analysis of GABRD protein expression in gastric adenocarcinoma versus normal gastric tissues. (D) GABRD protein expression in 86 paired samples of gastric adenocarcinoma and adjacent normal tissues. (E) Percentage of high‐level GABRD expression in cancerous versus normal tissues. (F) Correlation between GABRD expression and selected clinicopathological characteristics (T category, N category, and stage grouping) in gastric cancer patients. Subgroups with extremely small sample sizes—T1 (*n* = 2) and stage IV (*n* = 1)—were excluded from the statistical analysis. ns: *p* > 0.05, *: *p* <= 0.05, **: *p* <= 0.01, ***: *p* <= 0.001, ****: *p* <= 0.0001. Complete data are presented in Figure S1. (G) Representative immunohistochemical staining images of GABRD in different stages of gastric cancer and normal tissues. (H) Kaplan–Meier plots showing the association between GABRD protein expression levels and overall survival (OS) in gastric cancer patients. (I) Multivariate Cox regression analysis demonstrating GABRD expression as an independent prognostic factor for patient survival.

To explore the potential roles of GABRD in the carcinogenesis of gastric cancer, we first analysed the GABRD mRNA expression levels in 375 stomach adenocarcinoma tissues and 32 normal tissues from the TCGA database. The tumour tissues exhibited significantly higher GABRD mRNA expression levels than the normal tissues (Figure 1A). This result was also confirmed in 27 paired gastric cancer tissues and adjacent tumour tissues (Figure 1B). Then, we performed immunohistochemical (IHC) analysis to detect GABRD protein expression on the tissue micro‐array of 86 pairs of gastric adenocarcinoma tissues and normal gastric tissues plus 8 gastric cancer tissues (HStmA180Su19). The results indicated that GABRD protein was over‐expressed in gastric cancer samples versus normal samples (Figure 1C,D), with high GABRD in 49% of cancerous tissues versus 3.5% in adjacent para‐carcinoma tissues (Figure 1E). We then evaluated the potential associations between GABRD expression and clinicopathological characteristics of gastric cancer patients. GABRD protein expression levels were significantly correlated with the T category, N category, and stage grouping (Figure 1F; Figure S1; Table S4), confirmed by the Spearman correlation analysis (Table S5). Representative pictures of staining degrees of GABRD between different stage tumour tissue and normal tissue were shown in Figure 1G. Kaplan–Meier plots showed that the high level of GABRD protein expression was associated with lower overall survival (OS) (Figure 1H). The multivariate Cox regression analysis showed that GABRD expression was an independent prognostic factor for patient survival (HR = 2.14, *p* = 0.025), in addition to T category (Figure 1I; Table S6). These findings suggest that high expression of GABRD is significantly associated with poor clinical outcomes in gastric cancer, proposing GABRD as a potential oncogene.

### 3.2 GABRD Knockdown Inhibited Proliferation and Promoted Apoptosis in Gastric Cancer Cells

**FIGURE 2:**
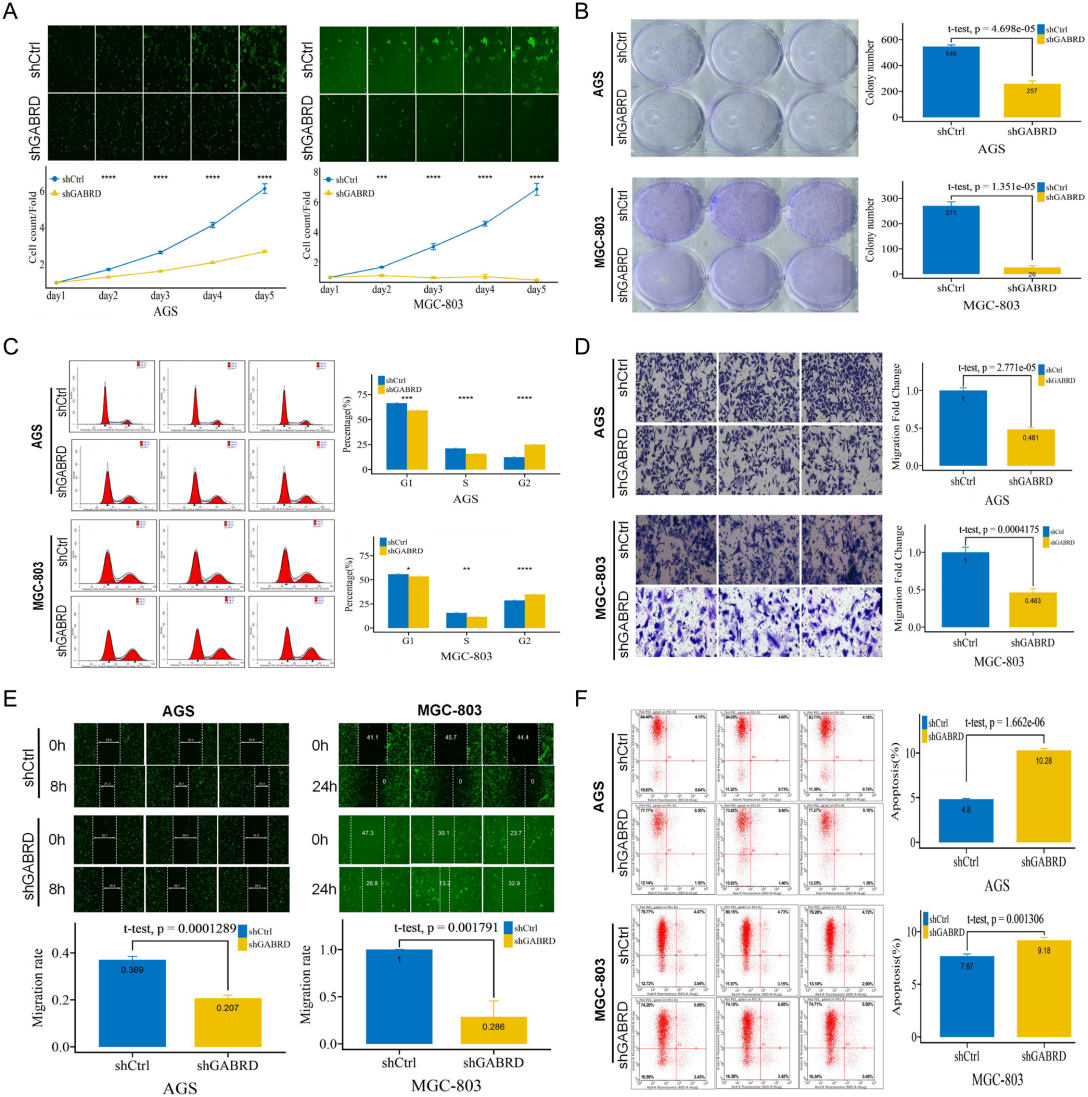
Impact of GABRD silencing on gastric cancer cell growth and apoptosis. (A) Celigo Cell Counting assay results show the effect of GABRD knockdown on the proliferation of AGS and MGC‐803 cell lines. ***: *p* <= 0.001, ****: *p* <= 0.0001. (B) Cell colony formation assay demonstrating the impact of GABRD knockdown on the clonal formation ability of AGS and MGC‐803 cell lines. (C) Alterations in cell cycle distribution induced by GABRD knockdown, as assessed by flow cytometry. *: *p* <= 0.05, **: *p* <= 0.01, ***: *p* <= 0.001, ****: *p* <= 0.0001. (D) Transwell migration assay results illustrate the effect of GABRD knockdown on cell migration in AGS and MGC‐803 cell lines. (E) Wound‐healing assay showing the impact of GABRD knockdown on cell invasion in AGS and MGC‐803 cell lines. (F) Annexin V‐APC staining and flow cytometry analysis indicating the percentage of apoptotic cells in AGS and MGC‐803 cell lines following GABRD knockdown.

After evaluating GABRD expression in gastric cancer and normal cell lines, AGS and MGC‐803 cells were chosen for further investigation due to their higher and stable GABRD levels (Figure S2A). GABRD was knocked down by small hairpin RNA (shRNA) targeting GABRD to explore the potential role of GABRD in gastric cancer cells. The results of qRT‐PCR showed that the mRNA of GABRD was significantly down‐regulated after shGABRD transfection (Figure S2B). In addition, the successful knockdown of GABRD was also verified by Western blot (Figure S2C,D). The Celigo Cell Counting assay showed GABRD knockdown markedly impeded proliferation in gastric cancer cells (Figure 2A). The cell colony formation experiment confirmed that the clonal formation ability decreased significantly in the shGABRD group than in the shCtrl group (Figure 2B). These results suggested that GABRD can promote the proliferation of gastric cancer cells. Consistent with this observation, the knockdown of GABRD affected cell cycle distribution and induced sub‐G2 phase arrest (Figure 2C). Then, the effect of GABRD on gastric cancer cell migration and invasion was estimated by transwell migration and wound‐healing assays. We found knockdown of GABRD expression also profoundly suppressed cell migration and invasion (Figure 2D,E). Next, to examine the effect of GABRD on cell apoptosis, Annexin V‐APC staining showed that the percentage of apoptotic gastric cancer cells was remarkably elevated in the shGABRD group than in the shCtrl group (Figure 2F). These results highlight GABRD's oncogenic potential in gastric cancer by enhancing cell proliferation, migration, and invasion, and inhibiting apoptosis.

### 3.3 GABRD Promoted the Tumorigenic Behaviour of Gastric Cancer Cells In Vivo

**FIGURE 3:**
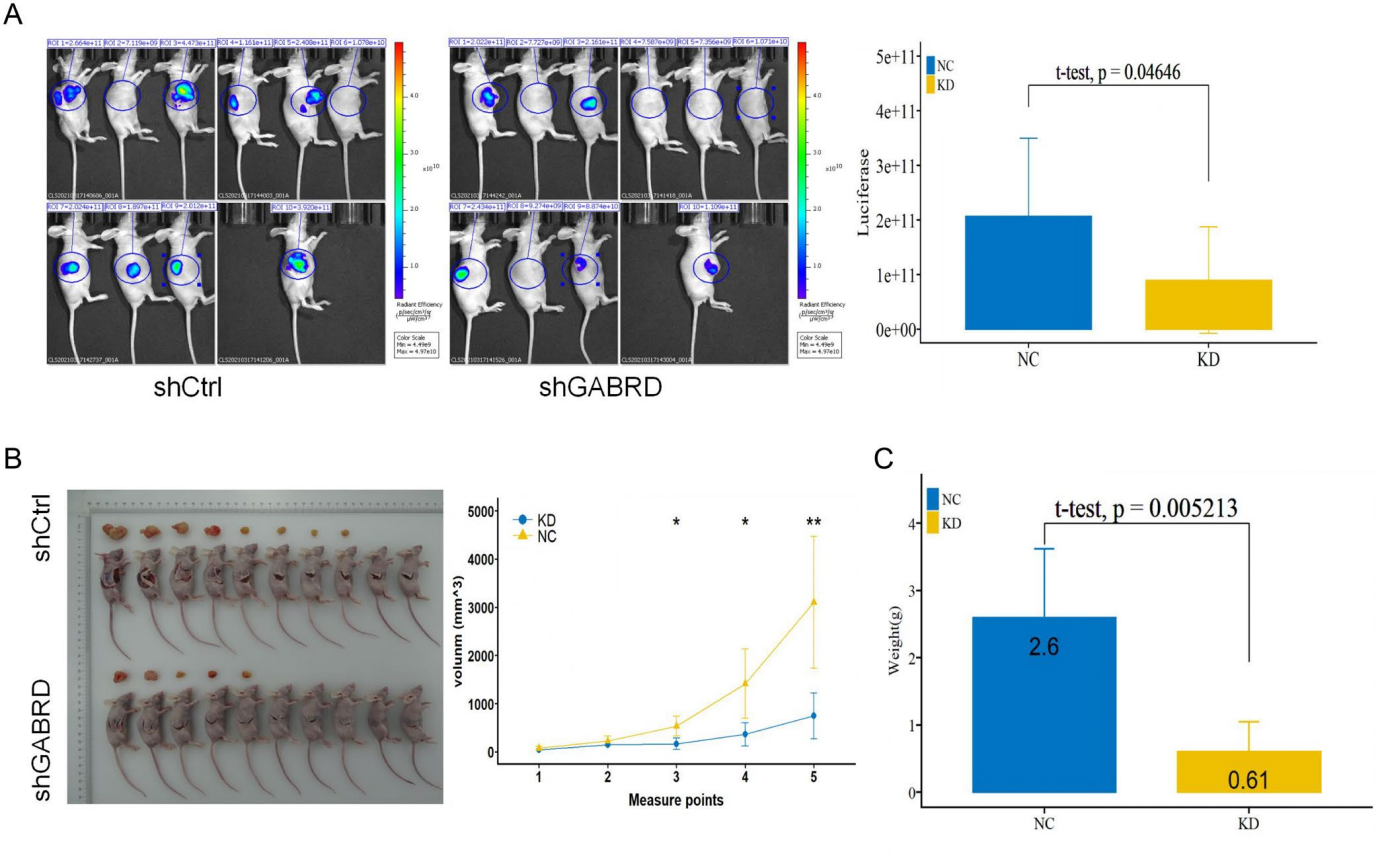
Investigation of GABRD's role in enhancing gastric cancer cell tumorigenicity in vivo. (A) In vivo bioluminescence imaging reflecting tumour size in mice injected with MGC‐803 cells treated with either shGABRD or shCtrl. (B) Measurement of tumour volume in mice injected with shGABRD or shCtrl‐treated MGC‐803 cells. *: *p* <= 0.05, **: *p* <= 0.01. (C) Comparison of average tumour weight between mice injected with shGABRD or shCtrl‐treated MGC‐803 cells.

To further explore whether GABRD plays a role in gastric cancer development in vivo, a xenograft model was established based on nude mice through subcutaneous injection of MGC‐803 cells treated with shGABRD or shCtrl, and tumour growth was subsequently quantified. Results of in vivo bioluminescence imaging, indicative of tumour size, showed that down‐regulation of the expression of GABRD could inhibit the development of gastric cancer in mice (Figure 3A). Tumour volume measurements in mice demonstrated that the shGABRD group showed a slower increase in tumour volume compared with the shCtrl group (Figure 3B). Knockdown of GABRD resulted in a decrease in the mean weight of tumours (Figure 3C). Together, these data further suggested that GABRD significantly promotes gastric cancer cell tumorigenesis and growth in vivo.

### 3.4 GABRD's Influence on Gene Expression and Identification of Crucial Downstream Molecules

**FIGURE 4:**
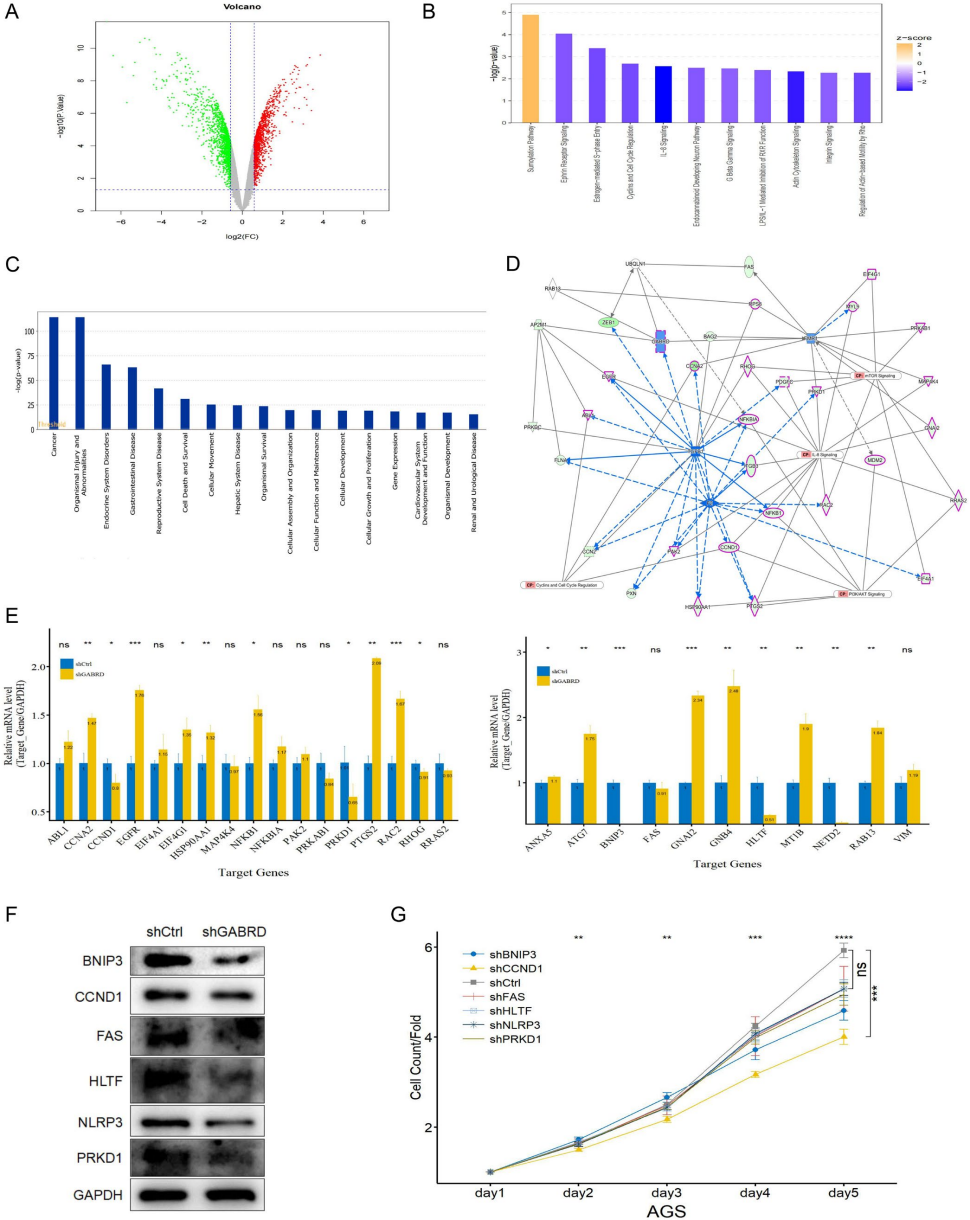
Exploring GABRD's influence on gene expression and identification of key downstream targets in gastric cancer. (A) Affymetrix Clariom S human assay results showing gene expression alterations in AGS cells following GABRD silencing. (B) Ingenuity Pathway Analysis (IPA) depicting significant alterations in key signalling pathways influenced by GABRD. (C) Schematic representation of the impact of GABRD silencing on cancer‐related pathways, highlighting Ephrin Receptor and IL‐8 signalling. (D) Protein–protein interaction (PPI) network analysis reveals potential regulation of critical oncogenic pathways by GABRD. (E and F) Results of qPCR and Western blot analyses confirming the downregulation of key genes following GABRD knockdown in AGS cells. (G) Functional significance of downregulated genes demonstrated by the Celigo Cell Counting assay. *: *p* <= 0.05, **: *p* <= 0.01, ***: *p* <= 0.001, ****: *p* <= 0.0001.

The crucial involvement of GABRD in gastric cancer progression led us to explore GABRD‐regulated genes. By employing the Affymetrix Clariom S human assay on AGS cells, 2644 genes were identified as influenced by GABRD: 1199 were upregulated and 1445 downregulated following GABRD suppression (GEO: GSE248437, Figure 4A). We further analysed the differentially expressed genes using QIAGEN's Ingenuity Pathway Analysis (IPA) software, which yielded insights into canonical pathways, upstream regulators, and interaction networks. Our core analysis indicated a significant overlap of these genes with the downregulation of pathways including Ephrin Receptor Signalling, Oestrogen‐mediated S‐phase Entry, Cyclins and Cell Cycle Regulation, and IL‐8 signalling (Figure 4B). IPA highlighted the top 5 impacted disease or function categories as cancer, organismal injury and abnormalities, endocrine system disorders, and gastrointestinal diseases (Figure 4C). Focusing on significantly enriched pathways like Cyclins and Cell Cycle Regulation and IL‐8 signalling, along with the classical cancer pathways of PI3K/AKT Signalling and mTOR Signalling, we conducted a protein–protein interaction (PPI) network analysis. This investigation aimed to unravel the critical molecular mechanisms GABRD employs in tumorigenesis regulation. The findings suggest GABRD, through intermediaries such as FMR1 and AP2M1, could influence the regulation of key genes including ABL1, CCNA2, CCND1, and others within these pathways (Figure 4D). Moreover, validation through qPCR and Western blot analyses in AGS cells revealed GABRD knockdown downregulated BNIP3, HLTF, NETD2, PRKD1, and CCND1 (Figure 4E,F). Functionally, the Celigo Cell Counting assay demonstrated that knocking down these genes significantly reduced AGS cell proliferation, especially CCND1 (Figure 4G). This evidence points toward CCND1 as a primary target for further molecular mechanism investigation.

### 3.5 The Function of GABRD in Regulating Proliferation, Migration, Invasion, and Apoptosis Depending on CCND1

**FIGURE 5:**
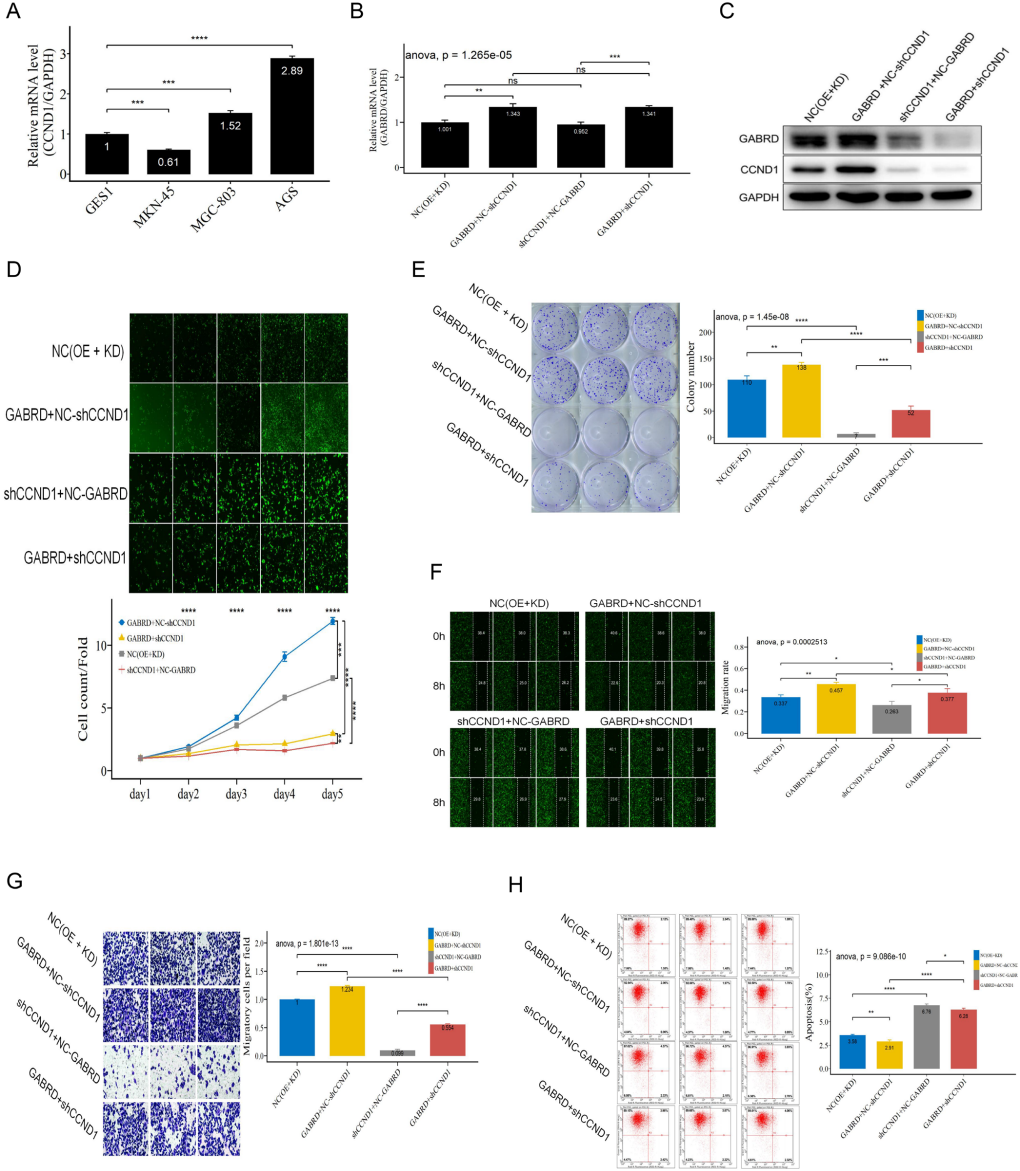
GABRD regulation of cell proliferation, migration, invasion, and apoptosis through CCND1. ns: *p* > 0.05, *: *p* <= 0.05, **: *p* <= 0.01, ***: *p* <= 0.001, ****: *p* <= 0.0001. (A) CCND1 expression levels in gastric cancer cell lines and a normal cell line (GES1), highlighting higher expression in AGS and MGC‐803 cells. (B) GABRD's impact on CCND1 expression validated by qRT‐PCR in AGS cells. (C) Western blot confirms GABRD's influence on CCND1 protein levels in AGS cells. (D) Celigo cell counting assays reveal the proliferative effects of CCND1 knockdown and GABRD overexpression in AGS cells. (E) Counteraction of GABRD‐induced proliferation by CCND1 knockdown in AGS cells, as demonstrated by colony formation assays. (F) Wound‐healing assays showing reversal of GABRD‐mediated cell migration promotion by CCND1 silencing in AGS cells. (G) Transwell assays confirming the attenuation of GABRD‐induced cell invasion by CCND1 knockdown in AGS cells. (H) Flow cytometry analysis illustrating the impact of CCND1 knockdown on GABRD‐mediated apoptosis in AGS cells.

To detect whether GABRD influences cancer cell proliferation and motility abilities via CCND1, we performed a series of rescued functional experiments. First, we analysed CCND1 expression levels in gastric cancer cell lines and a normal gastric mucosal epithelial cell line (GES1) by qRT‐PCR. The results showed that, compared with the GES1 cells, CCND1 is highly expressed in MGC‐803 and AGS cells (Figure 5A), choosing AGS for further tests due to its moderate CCND1 levels. Further, qRT‐PCR and Western blot analyses confirmed that GABRD upregulates CCND1, while CCND1 knockdown did not impact GABRD levels (Figure 5B,C). Therefore, CCND1 was a downregulated gene of GABRD, and subsequently, cell proliferation, migration, invasion, and apoptosis were assessed. The results of the Celigo cell counting assay and colony formation assay exhibited that, compared with the NC group, the proliferation and colony formation ability of AGS cells were significantly reduced by CCND1 knockdown and increased by GABRD overexpression, while a concomitant knockdown of CCND1 abolished the GABRD‐induced cell proliferation effects in AGS cells (Figure 5D,E). Meanwhile, CCND1 silencing also reversed GABRD's promotion of migration and invasion, as shown in wound‐healing and transwell assays (Figure 5F,G). As evaluated by flow cytometry, knockdown of CCND1 enhanced the apoptosis of AGS cells, and the inhibitory function on cell apoptosis caused by GABRD overexpression was rescued upon co‐knockdown of CCND1(Figure 5H). These data indicate a strong functional relationship between GABRD and CCND1, and CCND1 was necessary for proliferation, migration, invasion, and apoptosis.

### 3.6 Analysis of the Association and Physical Interaction Between GABRD and CCND1

**FIGURE 6:**
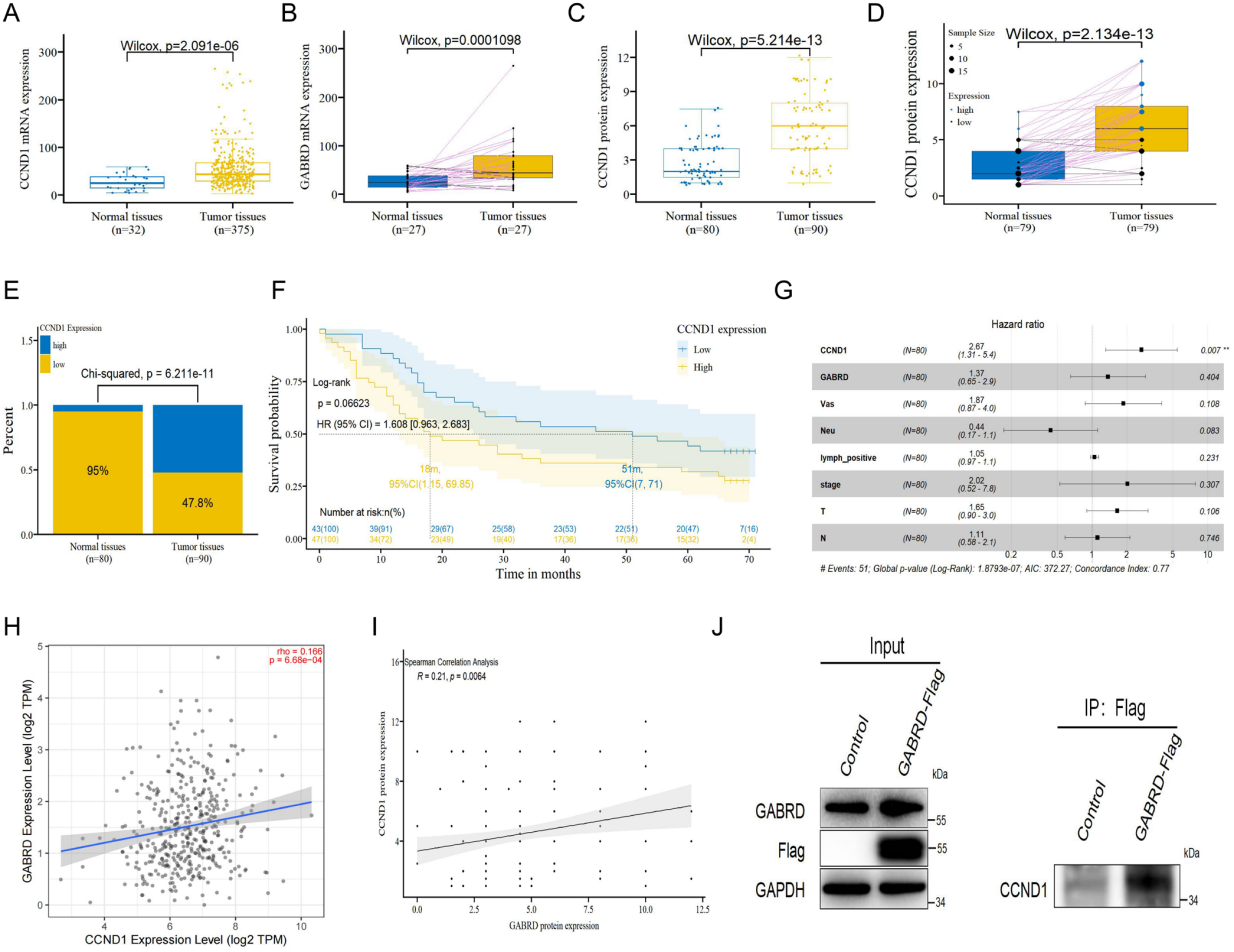
Correlation between CCND1 expression, prognosis, and association with GABRD in gastric cancer. (A) Differential CCND1 mRNA expression in gastric cancer versus normal tissues, highlighting elevated GABRD levels in tumour tissues. (B) Confirmation of elevated GABRD mRNA expression in tumour tissues compared to adjacent non‐tumour tissues. (C) Immunohistochemistry (IHC) reveals pronounced CCND1 protein overexpression in gastric cancer samples. (D) IHC validation of CCND1 protein overexpression in paired gastric cancer and normal tissues. (E) High CCND1 expression frequency in cancerous versus non‐cancerous tissues. (F) Kaplan–Meier survival curves indicate a trend toward poorer prognosis with higher CCND1 expression. (G) Multivariate Cox regression analysis establishing CCND1 as an independent prognostic indicator. (H) Correlation analysis between GABRD and CCND1 mRNA expression levels in TCGA‐STAD dataset, revealing a significant yet weak correlation. (I) Pearson's correlation confirms a significant association between GABRD and CCND1 protein expressions. (J) Co‐immunoprecipitation (Co‐IP) assay demonstrating physical interaction between CCND1 and GABRD in AGS cells.

Given the regulatory influence of GABRD on CCND1, we delved deeper into the interaction between GABRD and CCND1 to better understand their connection. TCGA‐STAD data revealed CCND1 mRNA levels significantly elevated in gastric cancer compared to normal tissues (Figure 6A), corroborated by analysis of 27 paired samples (Figure 6B). Immunohistochemistry (IHC) analysis on the tissue microarray also revealed significant CCND1 protein overexpression in cancer samples versus normal (Figure 6C,D), with high CCND1 expression in 52.2% of cancerous tissues versus 5% in adjacent para‐carcinoma tissues (Figure 6E). Kaplan–Meier survival analysis indicated that higher CCND1 protein expression correlated with poorer prognosis, although not statistically significant (*p* = 0.06623, HR = 1.608) (Figure 6F). However, multivariate Cox regression identified CCND1 as an independent prognostic factor for overall survival (HR = 2.673, *p* = 0.00671) (Figure 6G; Table S7). Further investigation into the GABRD and CCND1 interaction showed a significant, albeit weak, correlation between their mRNA expression levels in TCGA‐STAD (rho = 0.166, *p* = 6.68e‐04, Figure 6H). Additional IHC analysis confirmed a significant association between GABRD and CCND1 protein expressions in gastric cancer tissues (*p* = 0.00917, Figure 6I). A co‐immunoprecipitation assay demonstrated a physical interaction between CCND1 and GABRD in AGS cells, confirming their cooperative role in cancer progression (Figure 6J). These results highlight the intertwined roles of GABRD and CCND1 in promoting gastric cancer development and progression.

### 3.7 GABRD Knockdown Caused p53‐Dependent Apoptosis via CCDN1

**FIGURE 7:**
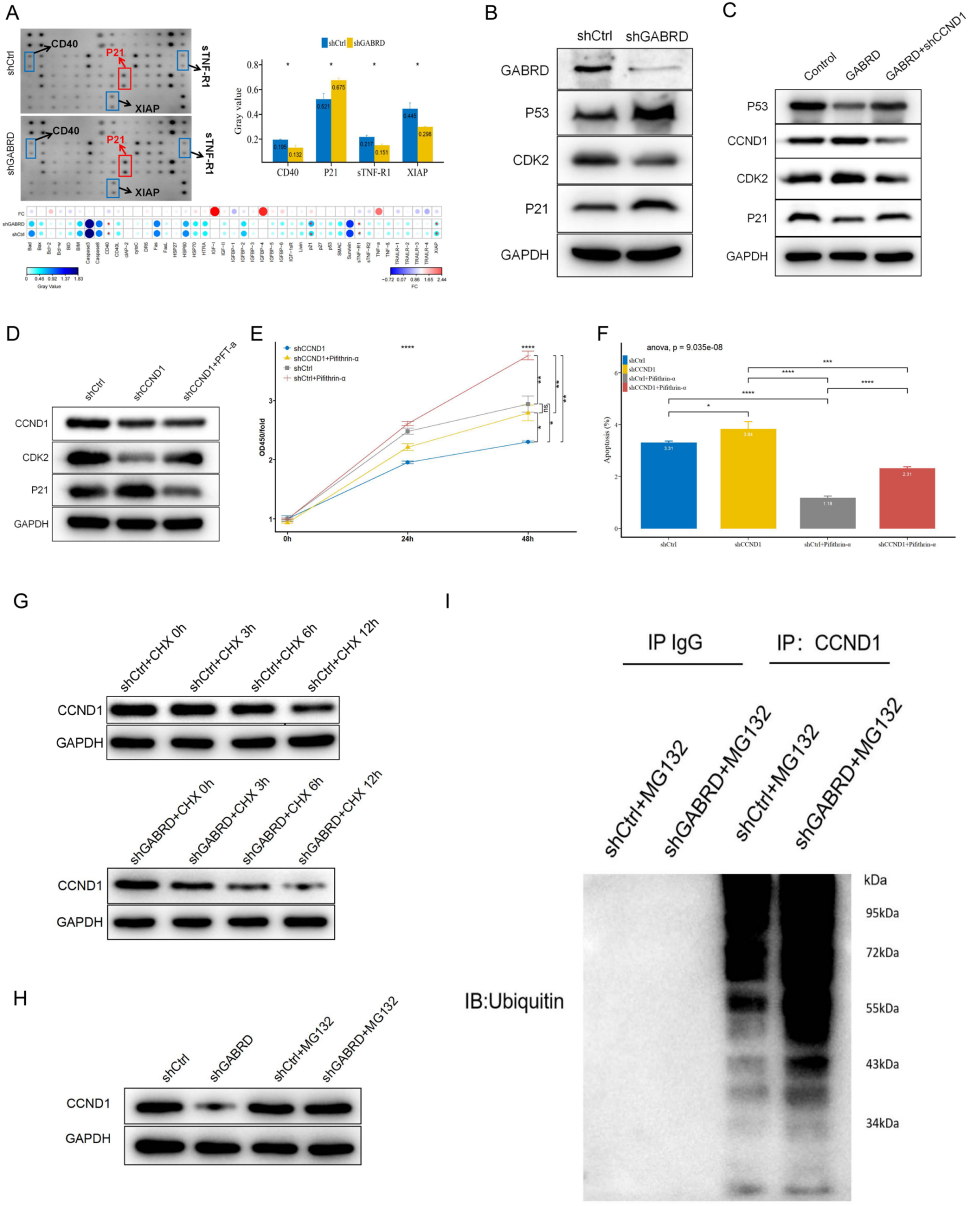
GABRD knockdown induces p53‐dependent apoptosis through CCND1 and GABRD stabilises CCND1 via inhibiting its ubiquitin‐mediated degradation. ns: *p* > 0.05, *: *p* <= 0.05, **: *p* <= 0.01, ***: *p* <= 0.001, ****: *p* <= 0.0001. (A) Human Apoptosis Antibody Array results showing alterations in apoptosis‐related protein expression in AGS cells following GABRD knockdown. (B) Western blot analysis demonstrating the effect of GABRD knockdown on p53 expression in AGS cells. (C) Western blot analysis showing the impact of CCND1 knockdown on p53 expression in AGS cells. (D) Inhibition of p53 negating the induction of downstream p53 genes by shGABRD or shCCND1. (E) CCK‐8 assay results indicate the impact of pifithrin‐α on the apoptosis‐promoting effect of GABRD knockdown in AGS cells. (F) Annexin V‐APC staining demonstrating the effect of pifithrin‐α on apoptosis induction following GABRD knockdown in AGS cells. (G) Cycloheximide (CHX) treatment reveals accelerated CCND1 degradation in GABRD‐knockdown AGS cells. (H) Proteasome inhibitor MG132 rescues GABRD‐knockdown‐induced CCND1 degradation. (I) In vitro ubiquitination assay indicates increased CCND1 ubiquitination upon GABRD knockdown.

To delve deeper into the changes in apoptosis‐related protein expression in GABRD‐induced AGS cells, we utilised a Human Apoptosis Antibody Array. The results indicated a significant increase in the expression of p21 (CDKN1A) in the shGABRD group, while the levels of three other proteins, CD40, sTNF‐R1, and XIAP, were reduced (Figure 7A). Considering p21 is regulated by p53 and CCND1 influences apoptosis through p53, it leads to the hypothesis that GABRD could potentially interfere with the p53 pathway in gastric cancer. Western blotting revealed GABRD knockdown elevated p53 levels (Figure 7B), and CCND1 suppression increased p53, partially countering GABRD's p53 inhibition (Figure 7C). Pifithrin‐α, a p53 inhibitor, negated the upregulation of p53 target genes by shGABRD or shCCND1 (Figure 7D). Further experiments assessing the effect of the CCND1/p53 axis on apoptosis via CCK‐8 assays and Annexin V‐APC staining showed that pifithrin‐α completely reversed the apoptosis induced by GABRD knockdown (Figure 7E,F). These results highlight the importance of the GABRD‐CCND1‐p53 pathway in apoptosis regulation, underscoring its potential as a therapeutic target in gastric cancer treatment.

### 3.8 GABRD Upregulated CCDN1 Through Inhibiting Its Ubiquitin‐Mediated Degradation

To delve deeper into the interaction between GABRD and CCND1, particularly in terms of CCND1's stability, the effect of GABRD on CCND1 degradation was examined using cycloheximide (CHX), a protein synthesis inhibitor. Post‐CHX treatment, CCND1 levels downregulated more in cells with GABRD knockdown compared to controls, hinting at GABRD's role in slowing CCND1 degradation, thus maintaining its cellular stability and abundance (Figure 7G). Subsequent experiments with the proteasome inhibitor MG132 revealed that proteasome blockade could prevent the enhanced degradation of CCND1 observed with GABRD knockdown (Figure 7H), suggesting GABRD protected CCND1 from proteasome‐driven destruction. An in vitro ubiquitination assay further revealed that GABRD knockdown significantly elevated the ubiquitination of CCND1 (Figure 7I), strengthening the notion that GABRD's interaction with CCND1 safeguards it from ubiquitination and the subsequent proteasomal degradation. These findings collectively underscore the critical role of GABRD in stabilising CCND1, thus contributing to its dysregulation and the proliferation of cancer cells.

## 4 Discussion

The growing interest in GABAergic signalling highlights the roles of GABA beyond its primary function as the inhibitory neurotransmitter of the central nervous system [7]. Notably, GABA serves as a metabolite in the Krebs cycle, underscoring its potential impact on processes beyond neurological disorders, including various cancers [6, 10]. Recent studies have observed increased GABA receptor expression in several cancer types, such as breast [6], colon [14], stomach [11], pancreas [10], adrenocortical carcinoma [18], and chondrosarcoma [3]. Intriguingly, analyses of TCGA data reveal that GABRD, encoding a GABA receptor subunit, is often upregulated in tumours compared to adjacent normal tissues [12]. This upregulation has been associated with poorer survival outcomes in colon adenocarcinoma, and GABRD is an independent prognostic factor [14, 15, 19]. However, the detailed biological functions and molecular mechanisms of GABRD in cancer development and progression have not been fully elucidated.

Our investigation revealed significant GABRD overexpression in both gastric cancer tissues and cell lines, aligning with analyses from the TCGA‐Stomach Adenocarcinoma (TCGA‐STAD) dataset. This consistency across studies reinforces the presence of a GABAergic signalling system within gastrointestinal cancer cellsr [13, 14, 15, 16, 19]. Additionally, GABRD overexpression in our study was significantly associated with advanced tumour features such as lymph node metastasis and higher T‐stage, indicating an association between GABRD levels and the metastatic potential of gastric cancers. However, due to the limited number of T1‐stage tumours in our dataset (only two cases) and the lack of metastatic samples (only one case), we were unable to detect a statistically significant difference in GABRD expression between T1 and T2–4 stages, nor confirm any correlation with distant metastasis (Figure S1). These sample constraints underscore the need for larger, more detailed studies to fully evaluate the role of GABRD expression across the full clinical spectrum of gastric cancer.

We identified GABRD as a novel oncogene acting through the CCND1/p53 axis in GC. CCND1 is associated with cancer development and is frequently overexpressed in various human cancer cells and tissues, including gastric cancer [20, 21]. As an oncogene that promotes cell cycle progression, high CCND1 expression is related to increased cancer cell growth and proliferation and decreased cell apoptosis [20]. Several reports have shown that CCND1 overexpression is associated with shorter survival in patients with gastric cancer [20, 21, 22]. A potential relationship between GABAergic signaling and CCND1 in gastric cancer has also been reported in the literature [11]. This study demonstrated that the proliferative effects of GABA in KATO III cells are associated with the upregulation of cyclin D1 (CCND1) expression [11]. However, it is not clear whether the GABA receptor regulates CCND1 in cancer. In this study, we demonstrated that GABRD directly regulates CCND1 expression by physical interaction, and the function of GABRD in promoting proliferation and migration relied on CCND1. To the best of our knowledge, the present study is the first to elucidate the strong relationship between GABRD and CCND1. In our present study, we found that high CCND1 expression levels are correlated with shorter overall survival of gastric cancer patients. CCND1, a key factor promoting cancer cell cycle progression, survival, and proliferation, is a typical substrate of the ubiquitin‐proteasome pathway. Thus, the periodicity and turnover of CCND1 are mainly determined by ubiquitin‐mediated proteolysis. Recent studies showed that some deubiquitinases interacted with CCND1 and prevented its polyubiquitination and degradation, resulting in cell cycle progression and cancer cell growth [23, 24, 25].

Our findings identify GABRD as a novel oncogene in gastric cancer, promoting malignant phenotypes through CCND1‐dependent cell cycle regulation. GABRD knockdown significantly suppresses tumour proliferation and migration in vitro and in vivo, and its upregulation correlates with advanced tumour stage and unfavourable prognosis. Notably, these results fit within the broader paradigm of neurotransmitter signalling in cancer, wherein intratumoral nerves and neurotransmitters have been shown to facilitate tumour growth. Recent evidence demonstrates that functional neuronal circuits form within tumour microenvironments, exhibit higher electrical activity than normal tissues, and support cancer progression, potentially through active synapse‐like connections or paracrine neuromodulators [26]. Similarly, GABAergic signalling has been implicated in melanoma initiation, where GABA secreted by melanoma cells can inhibit electrical activity in co‐cultured keratinocytes, ultimately promoting oncogenic competence [27, 28]. Although our present study focuses primarily on GABRD's molecular interactions with CCND1 to elucidate the proliferative and pro‐tumorigenic effects observed with GABRD overexpression, these emerging reports underscore the potential importance of electrical activity and ion flux in tumorigenesis. It is plausible that the GABRD's function in gastric cancer may not be limited to intracellular protein–protein interactions but could also involve ion channel activity influencing membrane potentials or crosstalk with the tumour microenvironment. Future experiments employing pharmacological inhibition or electrophysiological recording of GABRD‐containing receptors will be instrumental in defining whether such electrical mechanisms contribute to gastric cancer progression. By situating GABRD within the growing field of neurotransmitter‐driven oncogenesis, we provide foundational evidence for further mechanistic investigations, including possible electrophysiological and microenvironmental interventions in gastric cancer. Clinical evaluations of GABRD and its downstream effectors may eventually enable precision therapies that target the aberrant GABAergic circuitry in the tumour milieu.

Another important aspect to consider is the subcellular localisation of delta‐containing receptors. Although GABA_A receptors are classically considered to function at the plasma membrane, our analyses were performed using total cellular lysates, and the observed regulation of the nuclear protein Cyclin D1 suggests that, within the context of the GABRD‐CCND1 axis, GABRD may function from intracellular pools (e.g., cytoplasmic and/or nuclear compartments). It is conceivable that dynamic trafficking or compartmentalisation of GABRD contributes to its oncogenic function in gastric cancer. Future studies employing subcellular fractionation and immunofluorescence imaging will be essential to determine the precise localisation of GABRD and its potential compartment‐specific functions.

Recent studies have also highlighted the role of GABA_A receptor auxiliary subunits, such as Clptm1 and TMEM132B, in modulating receptor function and cell signalling [29, 30]. Our preliminary in silico analysis of TCGA‐STAD data did not reveal significant mRNA‐level differences (*p* > 0.01) between tumour and normal tissues for these auxiliary subunits (data not shown), although transcriptional changes may not mirror protein‐level abundance or activity. Their potential involvement in modulating the activity of delta‐containing receptors could add another layer of complexity to GABRD‐mediated oncogenic signalling. Investigating the expression and interaction of Clptm1, TMEM132B, and other auxiliary subunits may provide further insights into how GABAergic signalling is integrated into the broader oncogenic network. Future research should address whether alterations in these auxiliary subunits contribute to the dysregulation of GABA_A_ receptor signalling in gastric cancer and whether they might serve as additional therapeutic targets.

## 5 Conclusions

Taken together, our study identifies GABRD as a potential oncogene in gastric cancer, promoting tumour progression and affecting patient survival outcomes. GABRD may be involved in malignant biological behaviour in GC by regulating CCND1 pathways. GABRD might be a novel prognostic biomarker and a therapeutic target for GC. While our work lays a foundation for understanding the clinical and molecular significance of GABRD in gastric cancer, it also opens several avenues for future research. Specifically, further investigations into the role of receptor electrical activity, subcellular localisation dynamics, and the involvement of other auxiliary subunits will be critical to fully elucidate the multifaceted roles of GABAergic receptors in tumour biology. We believe that these studies will not only deepen our understanding of gastric cancer pathogenesis but also pave the way for the development of novel therapeutic strategies targeting the GABAergic signalling pathway.

## Author Contributions

**Weibing Leng:** conceptualization (lead), data curation (equal), formal analysis (equal), funding acquisition (equal), investigation (equal), methodology (equal), project administration (lead), writing – original draft (lead), writing – review and editing (equal). **Jun Ye:** methodology (supporting), software (supporting), validation (supporting). **Zhenpeng Wen:** conceptualization (supporting), data curation (supporting), formal analysis (supporting), methodology (supporting), project administration (supporting), validation (supporting), writing – original draft (equal). **Han Wang:** data curation (supporting), formal analysis (supporting), project administration (supporting). **Zhenyu Zhu:** conceptualization (supporting), funding acquisition (supporting), investigation (supporting), project administration (supporting). **Xilin Song:** conceptualization (supporting), funding acquisition (supporting), investigation (supporting), project administration (supporting). **Kai Liu:** conceptualization (equal), data curation (equal), formal analysis (equal), funding acquisition (equal), investigation (equal), methodology (equal), project administration (equal), supervision (equal), writing – original draft (equal), writing – review and editing (equal).

## Ethics Statement

This study has been approved by the Ethics Committee of Sichuan University West China Hospital (2021–1224). All procedures involving mice were approved by the Experimental Animal Welfare Ethics Committee of Sichuan University West China Hospital (approval No. 2021587A).

## Consent

The authors have nothing to report.

## Conflicts of Interest

The authors declare no conflicts of interest.

## Supporting information


**Figure S1.** Correlation between GABRD expression and selected clinicopathological characteristics (T category and stage grouping) in gastric cancer patients with complete data.

**Figure S2.** GABRD Knockdown and Validation in Gastric Cancer Cells. (A) Initial assessment of GABRD expression in gastric cancer cell lines and normal epithelial cells. (B) Design and implementation of shGABRD lentivirus‐mediated knockdown in gastric cancer cells. (C) Confirmation of successful GABRD knockdown in AGS and MGC‐803 cells. (D) Validation of GABRD knockdown by Western blot analysis in AGS and MGC‐803 cells.

**Table S1.** Antibodies used in western blotting and immunohistochemistry (IHC).

**Table S2.** Target sequences used for gene knockdown.

**Table S3.** Primers used in RT‐qPCR.

**Table S4.** Association of GABRD protein expression with gastric cancer tumour characteristics.

**Table S5.** Spearman correlation between GABRD expression and clinical parameters in gastric cancer.

**Table S6.** Univariate and multivariate analyses assessing the impact of GABRD expression on overall survival in gastric cancer patients.

**Table S7.** Univariate and multivariate analyses assessing the impact of CCND1 expression on overall survival in gastric cancer patients.

## Data Availability

The datasets generated and analysed during the current study are available in the Gene Expression Omnibus (GEO) [https://www.ncbi.nlm.nih.gov/geo/query/acc.cgi?acc=GSE248437].
