# Supplementary figures and images for: GABRD Accelerates Tumour Progression via Regulating CCND1 Signalling Pathway in Gastric Cancer
Source: J Cell Mol Med. 2025 Mar 27;29(7):e70485. doi: 10.1111/jcmm.70485 (PMC11947670; doi:10.1111/jcmm.70485)

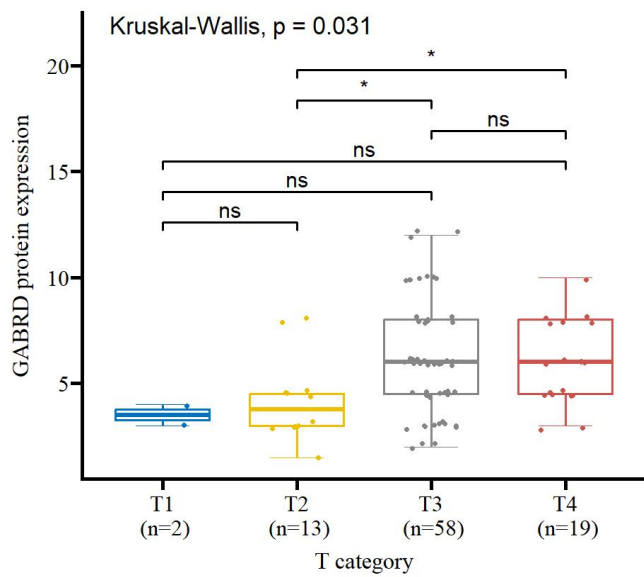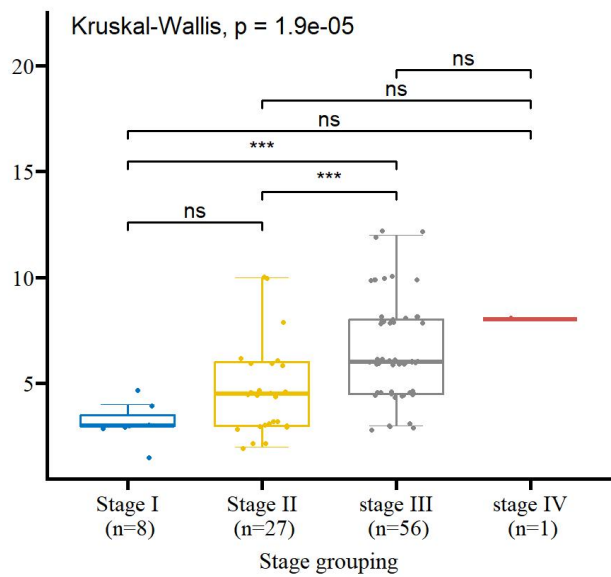

Supplement: Supplementary file 1 — Figure S1. Correlation between GABRD expression and selected clinicopathological characteristics (T category and stage grouping) in gastric cancer patients with complete data. [file JCMM-29-e70485-s006.pdf]

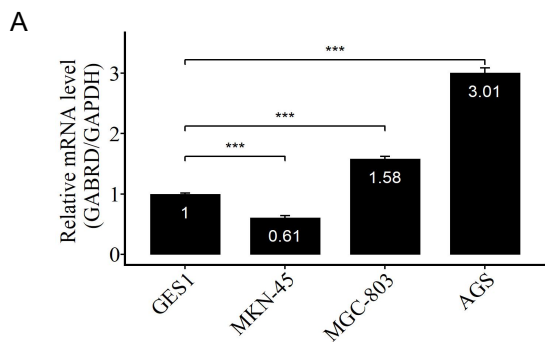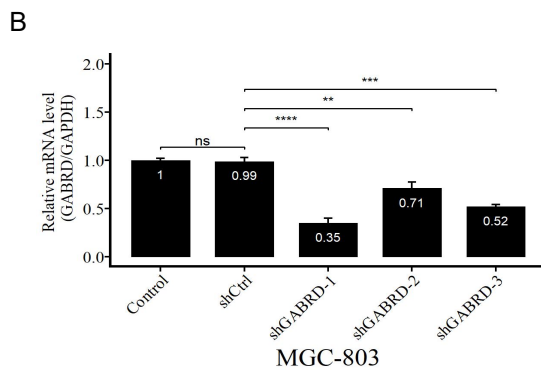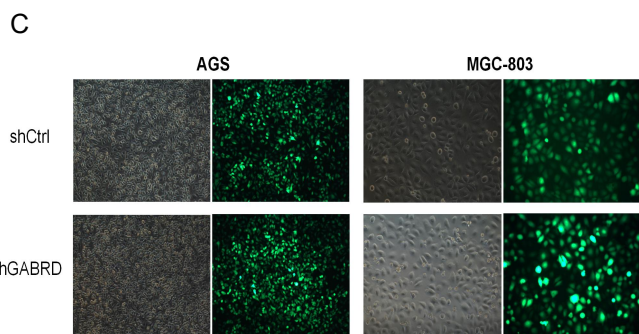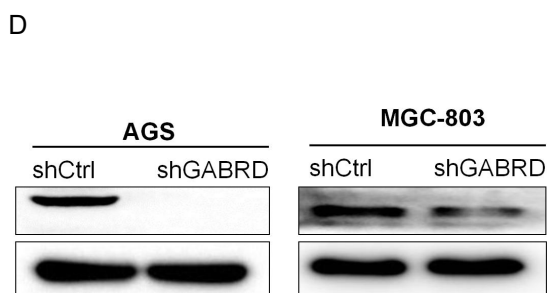

Supplement: Supplementary file 2 — Figure S2. GABRD Knockdown and Validation in Gastric Cancer Cells. (A) Initial assessment of GABRD expression in gastric cancer cell lines and normal epithelial cells. (B) Design and implementation of shGABRD lentivirus‐mediated knockdown in gastric cancer cells. (C) Confirmation of successful GABRD knockdown in AGS and MGC‐803 cells. (D) Validation of GABRD knockdown by Western blot analysis in AGS and MGC‐803 cells. [file JCMM-29-e70485-s007.pdf]
