# Supplementary material for: GABRD Accelerates Tumour Progression via Regulating CCND1 Signalling Pathway in Gastric Cancer
Source: J Cell Mol Med. 2025 Mar 27;29(7):e70485. doi: 10.1111/jcmm.70485 (PMC11947670; doi:10.1111/jcmm.70485)
Supplement: Supplementary file 3 — Table S1. Antibodies used in western blotting and immunohistochemistry (IHC). [file JCMM-29-e70485-s005.docx]

**Table S1.** Antibodies used in western blotting and immunohistochemistry (IHC).

| **Primary antibodies** | **Dilution in WB** | **Source species** | **Company** | **Catalog No.** |
| --- | --- | --- | --- | --- |
| GABRD | 1:1000 | Rabbit | thermo | PA5-26307 |
| AKT | 1:2000 | Mouse | systems | MAB2055-sp |
| P-AKT | 1:500 | Rabbit | systems | AF887-sp |
| CCND1 | 1:5000 | Rabbit | abcam | ab134175 |
| CDK6 | 1:1000 | Rabbit | abcam | ab151247 |
| PIK3CA | 1:1000 | Rabbit | abcam | ab40776 |
| GAPDH | 1:3000 | Rabbit | Bioworld | AP0063 |
| AKT | 1:2000 | Mouse | systems | MAB2055-sp |
| P-AKT | 1:500 | Rabbit | systems | AF887-sp |
| CCND1 | 1:5000 | Rabbit | abcam | ab134175 |
| GABRD | 1:1000 | Rabbit | thermo | PA5-26307 |
| CCND1 | 1:2000 | Rabbit | ABCAM | ab134175 |
| GAPDH | 1:30000 | Mouse | Proteintech | 60004-1-lg |
| P53 | 1:500 | Rabbit | Bioss | bs-8687R |
| CCND1 | 1:500 | Mouse | Proteintech | 60186-1-Ig |
| CDK2 | 1:1000 | Rabbit | CST | 2546 |
| P21 | 1:1000 | Rabbit | CST | 2947S |
| GABRD | 1:1000 | Rabbit | NOVUS | NB300-200 |
| P53 | 1:2000 | Rabbit | Proteintech | 10442-1-AP |

| **Primary antibodies** | **Dilution in Co-IP** | **Source species** | **Company** | **Catalog No.** |
| --- | --- | --- | --- | --- |
| DYKDDDDK Tag | 1:50/1:1000 | Rabbit | CST | 14793 |
| GABRD | 1:500 | Mouse | Santa Cruz | sc-271231 |
| CCND1 | 1:2000 | Rabbit | Abcam | ab134175 |
| GAPDH | 1:30000 | Mouse | Proteintech | 60004-1-lg |

| **Primary antibodies** | **Dilution in IHC** | **Source species** | **Company** | **Catalog No.** |
| --- | --- | --- | --- | --- |
| GABRD | 1:50 | Rabbit | NOVUS | NB300-200 |
| CCND1 | 1:100 | Rabbit | Bioss | bs-20596R |

| **Secondary antibody** | **Dilution** |  | **Company** | **Catalog No.** |
| --- | --- | --- | --- | --- |
| HRP Goat Anti-Rabbit IgG (WB and Co-IP) | 1:3000 |  | Beyotime | A0208 |
| HRP Goat Anti-Mouse IgG (WB and Co-IP) | 1:3000 |  | Beyotime | A0216 |
| HRP Goat Anti-Rabbit IgG (IHC) | 1:400 |  | Abcam | Ab97080 |
