# Supplementary material for: GABRD Accelerates Tumour Progression via Regulating CCND1 Signalling Pathway in Gastric Cancer
Source: J Cell Mol Med. 2025 Mar 27;29(7):e70485. doi: 10.1111/jcmm.70485 (PMC11947670; doi:10.1111/jcmm.70485)
Supplement: Supplementary file 4 — Table S2. Target sequences used for gene knockdown. [file JCMM-29-e70485-s002.docx]

**Table S2.** Target sequences used for gene knockdown.

| Gene symbol | Target sequence (5'-3') |
| --- | --- |
| GABRD-1 | CACCACGGAGCTGATGAACTT |
| GABRD-2 | CGACGTGACGGTGGAGAACAA |
| GABRD-3 | GGCAGAGATGGACGTGAGGAA |
| CCND1-1 | GGTGAACAAGCTCAAGTGGAA |
| CCND1-2 | CCACAGATGTGAAGTTCATTT |
| CCND1-3 | GGTGAACAAGCTCAAGTGGAA |
