# Supplementary material for: GABRD Accelerates Tumour Progression via Regulating CCND1 Signalling Pathway in Gastric Cancer
Source: J Cell Mol Med. 2025 Mar 27;29(7):e70485. doi: 10.1111/jcmm.70485 (PMC11947670; doi:10.1111/jcmm.70485)
Supplement: Supplementary file 5 — Table S3. Primers used in RT‐qPCR. [file JCMM-29-e70485-s008.docx]

**Table S3.** Primers used in RT-qPCR.

| Gene | Forward primer sequence (5'-3') | Reverse primer sequence (5'-3') |
| --- | --- | --- |
| GABRD | ATCGTGAACGCCAAGTCG | TGGAGGTGATTCGGATGCT |
| CCND1 | AGGCGGAGGAGAACAAACAGA | GGAGGGCGGATTGGAAATGAA |
| GAPDH | TGACTTCAACAGCGACACCCA | CACCCTGTTGCTGTAGCCAAA |
| FAS | GGACATGGCTTAGAAGTGGAAA | ACTTGGTGTTGCTGGTGAGTG |
| GNAI2 | GAACGACCTGGAGCGTATTG | TGTAGGTCCTTGAAGGTGAAGTG |
| RAB13 | CTTACATCTCCACCATCGG | TCCACGGTAGTAGGCAGT |
| BNIP3 | CTCTGCTGCTCTCTCATTTGC | CTTCATCAAAAGGTGCTGGTGG |
| ATG7 | ATGTGGAGCAACTGGAGC | GCAATGACGGCAGGAAGC |
| ANXA5 | CACCTGAAGAACTGAGAGCCAT | GAGAACCACCAACATCCGCT |
| VIM | GGTGCAATCGTGATCTGGGA | GTCTTTGCTCGAATGTGCGG |
| HLTF | ACACCACTGCTTCCACATCAA | TTCTGGTCGGTCCTTCTCAG |
| MT1B | CCTGCACCACAGGTGGCT | CAGCAAGAGCAGCAGCAC |
| GNB4 | CCAGCAGACCACCACATTCA | CAAGCACCAGAAACAAAAGTCC |
| NETD2 | GCATCTGGACCATTAAAGCCACT | CGAAACCTGCTAAGCCGACTAC |
| GNAI2 | GAACGACCTGGAGCGTATTG | TGTAGGTCCTTGAAGGTGAAGTG |
| RAB13 | CTTACATCTCCACCATCGG | TCCACGGTAGTAGGCAGT |
| BNIP3 | CTCTGCTGCTCTCTCATTTGC | CTTCATCAAAAGGTGCTGGTGG |
| ATG7 | ATGTGGAGCAACTGGAGC | GCAATGACGGCAGGAAGC |
| ANXA5 | CACCTGAAGAACTGAGAGCCAT | GAGAACCACCAACATCCGCT |
| ABL1 | ATCACGCCAGTCAACAGT | TCTCACGCACCAAGAAGC |
| NFKB1 | AGGATTTCGTTTCCGTTATGTATG | CCTGAGGGTAAGACTTCTTGTTCT |
| CCNA2 | AGCCTGCGTTCACCATTCA | GGGCATCTTCACGCTCTATTTT |
| NFKBIA | CTCCATCCTGAAGGCTACCAA | GCACCCAAGGACACCAAAAG |
| CCND1 | AGCTGTGCATCTACACCGAC | GAAATCGTGCGGGGTCATTG |
| PAK2 | TGCTGGATGTCCTAAAGTTCTAC | CTCTGTCACTACTGCGGGTG |
| EGFR | ATGAGGACATAACCAGCCACC | AGGCACGAGTAACAAGCTCAC |
| PRKAB1 | AATGGTGGATTCCCAAAAGTGC | TCAGGATGACCTGGAGGAGATG |
| EIF4A1 | TGGATGAAGCTGACGAAATG | CTGGGTGTTGCTGTTGAG |
| PRKD1 | TCTCGCCCCATCTTCCTCTAT | GAGGTAGGGTCATGGCGAAAA |
| EIF4G1 | CAGAATCCCAGCCTTCGTCG | AGACATTTGTATAGTTGTCATAGTGTCCC |
| PTGS2 | CAAATCCTTGCTGTTCCCACC | TTTCTCCATAGAATCCTGTCCG |
| RAC2 | TCGTCAGCCCAGCCTCTTAT | TCAGTTTCTCGATGGTGTCCTT |
| HSP90AA1 | TTGTAGACTGCCGAGTAATAGCC | TCCTCATCGCTGCCACTAA |
| RHOG | GGCGCACAGTGAACCTGAACCT | CTGGCAATGGAGAAACAGATGACG |
| RRAS2 | GAGGCATCAGCAAAGATTAGG | GAGGCATCAGCAAAGATTAGG |
| MAP4K4 | TGAGCAATGGTGAAACGGAA | TCTGGCGGACGATTAGAGTG |
| ABL1 | ATCACGCCAGTCAACAGT | TCTCACGCACCAAGAAGC |
| NFKB1 | AGGATTTCGTTTCCGTTATGTATG | CCTGAGGGTAAGACTTCTTGTTCT |
| CCNA2 | AGCCTGCGTTCACCATTCA | GGGCATCTTCACGCTCTATTTT |
|  |  |  |
