# Supplementary material for: GABRD Accelerates Tumour Progression via Regulating CCND1 Signalling Pathway in Gastric Cancer
Source: J Cell Mol Med. 2025 Mar 27;29(7):e70485. doi: 10.1111/jcmm.70485 (PMC11947670; doi:10.1111/jcmm.70485)
Supplement: Supplementary file 6 — Table S4. Association of GABRD protein expression with gastric cancer tumour characteristics. [file JCMM-29-e70485-s003.docx]

**Table S4.** Association of GABRD protein expression with gastric cancer tumor characteristics.

| Features | No. of patients | GABRD protein expression | | p value |
| --- | --- | --- | --- | --- |
|  |  | low | high |  |
| All patients | 94 | 48 | 46 |  |
| Age (years) |  |  |  | 0.220 |
|  | 94 | 48 | 46 |  |
| Gender |  |  |  | 0.427 |
| Male | 59 | 32 | 27 |  |
| Female | 35 | 16 | 19 |  |
| Tumor size |  |  |  | 0.681 |
| ≤4.7cm | 47 | 25 | 22 |  |
| ＞4.7cm | 47 | 23 | 24 |  |
| T [Infiltrate](D:/360%E5%AE%89%E5%85%A8%E6%B5%8F%E8%A7%88%E5%99%A8%E4%B8%8B%E8%BD%BD/Dict/8.4.0.0/resultui/html/index.html#/javascript:;) |  |  |  | 0.018* |
| T1 | 2 | 2 | 0 |  |
| T2 | 13 | 11 | 2 |  |
| T3 | 58 | 25 | 33 |  |
| T4 | 19 | 8 | 11 |  |
| Grade |  |  |  | 0.607 |
| 2 | 14 | 6 | 8 |  |
| 3 | 57 | 24 | 33 |  |
| 4 | 2 | 0 | 2 |  |
| [lymphatic](D:/360%E5%AE%89%E5%85%A8%E6%B5%8F%E8%A7%88%E5%99%A8%E4%B8%8B%E8%BD%BD/Dict/8.4.0.0/resultui/html/index.html#/javascript:;) [metastasis](D:/360%E5%AE%89%E5%85%A8%E6%B5%8F%E8%A7%88%E5%99%A8%E4%B8%8B%E8%BD%BD/Dict/8.4.0.0/resultui/html/index.html#/javascript:;)（N） |  |  |  | 0.001** |
| N0 | 22 | 17 | 5 |  |
| N1 | 11 | 6 | 5 |  |
| N2 | 23 | 13 | 10 |  |
| N3 | 38 | 12 | 26 |  |
| stage |  |  |  | 0.000*** |
| I | 8 | 8 | 0 |  |
| II | 27 | 19 | 8 |  |
| III | 56 | 19 | 37 |  |
| IV | 1 | 0 | 1 |  |
| Metastasis |  |  |  | 0.307 |
| 0 | 93 | 48 | 45 |  |
| 1 | 1 | 0 | 1 |  |
| Expession of PDL1 |  |  |  | 0.653 |
| 0 | 8 | 3 | 5 |  |
| 1.0 | 30 | 18 | 12 |  |
| 1.5 | 25 | 11 | 14 |  |
| 2.0 | 12 | 5 | 7 |  |
| 3.0 | 5 | 3 | 2 |  |
| 4.0 | 2 | 1 | 1 |  |
| 4.5 | 4 | 1 | 3 |  |
| 6.0 | 2 | 0 | 2 |  |
| 8.0 | 2 | 2 | 0 |  |
| 12.0 | 1 | 1 | 0 |  |
| Expession of MLH1 |  |  |  | 0.603 |
| 0. | 8 | 6 | 2 |  |
| 1.0 | 3 | 3 | 0 |  |
| 2.0 | 5 | 4 | 1 |  |
| 3.0 | 4 | 1 | 3 |  |
| 4.0 | 2 | 0 | 2 |  |
| 4.5 | 1 | 0 | 1 |  |
| 6.0 | 25 | 10 | 15 |  |
| 8.0 | 32 | 15 | 17 |  |
| 10.0 | 8 | 3 | 5 |  |
| 12.0 | 4 | 4 | 0 |  |
| Expession of PD1 |  |  |  | 0.959 |
| 0 | 7 | 6 | 1 |  |
| 0.5 | 11 | 3 | 8 |  |
| 1.0 | 71 | 35 | 36 |  |
| 2.0 | 1 | 1 | 0 |  |
| Expession of CD8 |  |  |  | 0.752 |
| 0 | 1 | 1 | 0 |  |
| 0.5 | 4 | 3 | 1 |  |
| 1.0 | 80 | 36 | 44 |  |
| 2.0 | 5 | 4 | 1 |  |
| 3.0 | 1 | 1 | 0 |  |
| Expession of Her2 |  |  |  | 0.372 |
| 0 | 88 | 46 | 42 |  |
| 1 | 6 | 2 | 4 |  |
