# Supplementary material for: GABRD Accelerates Tumour Progression via Regulating CCND1 Signalling Pathway in Gastric Cancer
Source: J Cell Mol Med. 2025 Mar 27;29(7):e70485. doi: 10.1111/jcmm.70485 (PMC11947670; doi:10.1111/jcmm.70485)
Supplement: Supplementary file 7 — Table S5. Spearman correlation between GABRD expression and clinical parameters in gastric cancer. [file JCMM-29-e70485-s001.docx]

**Table S5.** Spearman correlation between GABRD expression and clinical parameters in gastric cancer.

|  |  | GABRD |
| --- | --- | --- |
| Tumor Infiltrate | Spearman rank correlation coefficient | 0.453 |
|  | Significance (two-tails) | 0.000*** |
|  | N | 92 |
| lymphatic metastasis (N) | Spearman rank correlation coefficient | 0.351 |
|  | Significance (two-tails) | 0.001** |
|  | N | 94 |
| Stage | Spearman rank correlation coefficient | 0.248 |
|  | Significance (two-tails) | 0.017* |
|  | N | 92 |
