# Supplementary material for: GABRD Accelerates Tumour Progression via Regulating CCND1 Signalling Pathway in Gastric Cancer
Source: J Cell Mol Med. 2025 Mar 27;29(7):e70485. doi: 10.1111/jcmm.70485 (PMC11947670; doi:10.1111/jcmm.70485)
Supplement: Supplementary file 8 — Table S6. Univariate and multivariate analyses assessing the impact of GABRD expression on overall survival in gastric cancer patients. [file JCMM-29-e70485-s009.docx]

**Table S6.** Univariate and multivariate analyses assessing the impact of GABRD expression on overall survival in gastric cancer patients.

| Parameter | Univariate analysis | | | Multivariate analysis | | |
| --- | --- | --- | --- | --- | --- | --- |
|  | HR | 95% CI | *p* | HR | 95% CI | *p* |
| Sex | 1.043 | 0.595-1.83 | 0.882 |  |  |  |
| Age | 1.374 | 0.734-0.32 | 0.32 |  |  |  |
| Size | 1.569 | 0.912-2.7 | 0.104 |  |  |  |
| Vas | 2.845 | 1.627-4.973 | 0.000244 | 1.986 | 0.961-4.101 | 0.0638 |
| Neu | 1.987 | 1.017-3.883 | 0.0446 | 0.525 | 0.214-1.290 | 0.1600 |
| Lymph positive nodes | 1.111 | 1.066-1.159 | 7.89e-07 | 1.039 | 0.959-1.126 | 0.3450 |
| **T classification** | 2.167 | 1.372-3.424 | 0.000917 | 1.946 | 1.097-3.451 | **0.0228*** |
| N classification | 1.805 | 1.376-2.366 | 1.96e-05 | 1.393 | 0.740-2.621 | 0.3040 |
| Stage | 3.36 | 1.828-6.175 | 9.54e-05 | 0.831 | 0.263-2.625 | 0.7520 |
| **GABRD** | 3.152 | 1.736-5.724 | 0.000162 | 2.143 | 1.099-4.179 | **0.0252*** |
