# Supplementary material for: GABRD Accelerates Tumour Progression via Regulating CCND1 Signalling Pathway in Gastric Cancer
Source: J Cell Mol Med. 2025 Mar 27;29(7):e70485. doi: 10.1111/jcmm.70485 (PMC11947670; doi:10.1111/jcmm.70485)
Supplement: Supplementary file 9 — Table S7. Univariate and multivariate analyses assessing the impact of CCND1 expression on overall survival in gastric cancer patients. [file JCMM-29-e70485-s004.docx]

**Table S7.** Univariate and multivariate analyses assessing the impact of CCND1 expression on overall survival in gastric cancer patients.

| Parameter | Univariate analysis | | | Multivariate analysis | | |
| --- | --- | --- | --- | --- | --- | --- |
|  | HR | 95% CI | *p* | HR | 95% CI | *p* |
| Sex | 1.022 | 0.577-1.81 | 0.94 |  |  |  |
| Age | 1.363 | 0.727-2.555 | 0.334 |  |  |  |
| Size | 1.598 | 0.923-2.767 | 0.0943 |  |  |  |
| Vas | 2.884 | 1.643-5.061 | 0.000244 | 1.866 | 0.873-3.989 | 0.10800 |
| Neu | 1.996 | 1.019-3.909 | 0.0438 | 0.438 | 0.172-1.115 | 0.08330 |
| Lymph positive nodes | 1.114 | 1.068-1.162 | 6.15e-07 | 1.049 | 0.970-1.136 | 0.23100 |
| T classification | 2.168 | 1.37-3.433 | 0.000962 | 1.652 | 0.899-3.037 | 0.10600 |
| N classification | 1.864 | 1.407-2.469 | 1.44e-05 | 1.113 | 0.582-2.131 | 0.374600 |
| Stage | 3.54 | 1.877-6.677 | 9.43e-05 | 2.022 | 0.524-7.802 | 0.30700 |
| GABRD | 3.135 | 1.722-5.704 | 0.000184 | 1.374 | 0.652-2.895 | 0.40400 |
| **CCND1** | 1.796 | 1.023-3.154 | 0.0415 | 2.673 | 1.313-5.441 | **0.00671*** |
